# The potential biological activities of *Aspergillus luchuensis*-aided green synthesis of silver nanoparticles

**DOI:** 10.3389/fmicb.2024.1381302

**Published:** 2024-05-20

**Authors:** Rasha Y. Abd Elghaffar, Amany M. Emam, Ehab S. Taher, Mohamed M. Baz, Hamada Nayel, Ahmed Abdeen, Mohammad El-Nablaway, Khairiah M. Alwutayd, Ostan Mihaela, Banatean-Dunea Ioan, Abeer A. Khattab, Rasha H. Al‑Serwi, Amira E. Sehim

**Affiliations:** ^1^Department of Botany and Microbiology, Faculty of Science, Benha University, Benha, Egypt; ^2^Department of Basic Medical and Dental Sciences, Faculty of Dentistry, Zarqa University, Zarqa, Jordan; ^3^Department of Entomology, Faculty of Science, Benha University, Benha, Egypt; ^4^Department of Computer Science, Faculty of Computers and Artificial Intelligence, Benha University, Benha, Egypt; ^5^Department of Forensic Medicine and Toxicology, Faculty of Veterinary Medicine, Benha University, Toukh, Egypt; ^6^Department of Medical Biochemistry, Faculty of Medicine, Mansoura University, Mansoura, Egypt; ^7^Department of Basic Medical Sciences, College of Medicine, AlMaarefa University, Riyadh, Saudi Arabia; ^8^Department of Biology, College of Science, Princess Nourah bint Abdulrahman University, Riyadh, Saudi Arabia; ^9^Department of Biology, Faculty of Agriculture, University of Life Sciences "King Michael I" from Timisoara, Timisoara, Romania; ^10^Department of Basic Dental Sciences, College of Dentistry, Princess Nourah bint Abdulrahman University, Riyadh, Saudi Arabia

**Keywords:** endophytic fungi, biosynthetic metals, antimicrobial, antioxidant, dye degradation, larvicidal

## Abstract

Biosynthetic metals have attracted global attention because of their safety, affordability, and environmental friendliness. As a consequence, the cell-free filtrate (CFF) of Dill leaf-derived endophytic fungus *Aspergillus luchuensis* was employed for the extracellularly synthesis silver nanoparticles (AgNPs). A reddish-brown color shift confirmed that AgNPs were successfully produced. The obtained AgNPs were characterized by UV–Vis (ultraviolet–visible spectroscopy), Transmission electron microscopy (TEM), FTIR, EDX, and zeta potential. Results demonstrated the creation of crystalline AgNPs with a spherical shape at 427.81 nm in the UV–Vis spectrum, and size ranged from 16 to 18 nm as observed by TEM. Additionally, the biogenic AgNPs had a promising antibacterial activity versus multidrug-resistant bacteria, notably, *S. aureus*, *E. coli*, and *S. typhi*. The highest growth reduction was recorded in the case of *E. coli*. Furthermore, the biosynthesized AgNPs demonstrated potent antifungal potential versus a variety of harmful fungi. The maximum growth inhibition was evaluated from *A. brasinsilles*, followed by *C. albicans* as compared to cell-free extract and AgNO_3_. In addition, data revealed that AgNPs possess powerful antioxidant activity, and their ability to scavenge radicals increased from 33.0 to 85.1% with an increment in their concentration from 3.9 to 1,000 μg/mL. Furthermore, data showed that AgNPs displayed high catalytic activity of safranin under light irradiation. The maximum decolorization percentage (100%) was observed after 6 h. Besides, the biosynthesized AgNPs showed high insecticidal potential against 3^rd^ larval instar of *Culex pipiens*. Taken together, data suggested that endophytic fungus, *A. luchuensis,* is an attractive candidate as an environmentally sustainable and friendly fungal nanofactory.

## 1 Introduction

Nanotechnology science made it possible to find active substances that are applicable to numerous industries and fields including agriculture, biomedical (antimicrobial, antitumor, cytotoxicity, and cosmetics), textiles, heavy metal removal, wastewater treatment, medicine delivery, optoelectronics, and parasitology (Manimegalai et al., 2020; Hamza et al., 2021). Nanoparticles (NPs) have distinct physical, chemical, and structural characteristics, like their sizes, shapes, surface charges, stability, compatibility, and the proportion of their small size to their enormously surface area (Salem and Fouda, 2021). For the synthesis of nanomaterials, many researchers are encouraged to use metals, such as gold, silver, etc., owing to their physical and chemical properties (Castillo-Henríquez et al., 2020; Abinaya et al., 2021). However, the costs associated with the physical and chemical processes are higher, hazardous compounds are produced, and harsh synthesis conditions (such as temperature and pressure) are required (Alsharif et al., 2020). Due to these disadvantages, other biological sources including plants and microorganisms (fungi, yeast, bacteria, actinomycetes, and algae) have become widely used (Abdo et al., 2021). In this regard, biosynthesized NPs are utilized because they are environmentally friendly, have rapid effects, are economical, have a high degree of stability, and do not harm public health as much as chemical insecticides do (Athanassiou et al., 2018).

Interestingly, fungi are receiving greater focus than other microbes for producing various Nanoparticles of metallic and metallic oxides (Ammar et al., 2021; Alghuthaymi et al., 2022). This is because fungi can handle a lot of heavy metals, are easy to scale up, make a lot of biomasses, are easy to handle, and aren’t very toxic (Khalil et al., 2021). All these things make them good candidates for making NPs. In this context, Sagar and Ashok (2012) synthesized AgNPs from the supernatants of *A. niger*. They found that AgNPs were spherical polydispersed particles that varied in size between 1 and 20 nm and were stabilized in solution. In addition, AgNO_3_ was reduced by *A. flavus* F5 producing (AgNPs) which were verified through the creation of a yellowish-brown color (Fouda et al., 2022b). In addition, Majeed et al. (2018) use *Penicillium italicum* in order to produce AgNPs.

Biosynthesized AgNPs have been employed for their antimicrobial, antioxidative, anticancer, and larvicidal activities (Mistry et al., 2021). AgNPs and their ions have demonstrated efficacy as antibacterial agents versus numerous pathogenic bacteria by which the issue of multi-drug resistance is restricted (Salem et al., 2015; Gahlawat et al., 2016). Due to their size similarity, AgNPs penetrate cell walls and membranes, directly affecting intracellular components. Moreover, the antifungal potential of AgNPs has been assessed. Alhomaidi et al. (2022) showed that AgNPs from *Trichoderma harzianum* displayed antifungal potential versus *Macrophomina phaselina, Fusarium fujikuroi* and *Rhizoctonia solani* under greenhouse conditions. In addition, Xiao et al. (2023) documented the antifungal efficiency of AgNPs versus *Curvularia lunata.*

In recent years water pollution has become one of the most serious social problems as a result of the widespread use of dangerous chemicals such as organic dyes. In addition to being non-biodegradable, organic dyes pose a toxic effect on both human health and aquatic life. Dye wastes are considered the major environmental pollutants that are carcinogenic and mutagenic to humans (Manzoor and Sharma, 2020). Although both chemical and physical protocols have been established and used recently in order to remediate waste products, they have several disadvantages (Sabouri et al., 2021). Moreover, attempts to combat mosquitoes have gained great attention, particularly using eco-friendly agents such as green synthesized NPs (Athanassiou et al., 2018).

Based on the promising features and applications of AgNPs, this study was designed to screen the endophytic fungus *A. luchuensis* as a model biological system to synthesize AgNPs. Furthermore, the physical properties and potential applications of AgNPs as antibacterial, antioxidant, photocatalytic, and larvicidal agents were estimated.

## 2 Materials and methods

### 2.1 Microorganisms

*Aspergillus luchuensis* AUMC16034 strain was used for the synthesis of AgNPs. The fungal strain was isolated from the leaves of dill (Anethum graveolen) and identified morphologically and genetically. Moreover, multidrug-resistant bacteria, such as *Staphylococcus aureus* ATCC 25923, *Salmonella typhimurium* ATCC 6539, and *Escherichia coli* RCMB004001, in addition to pathogenic fungi, like *Fusarium oxysporum AUMC*15842, *Alternaria alternate* AUMC 15849, *Candida albicans* ATCC90028, *A. brasinsilles* AUMC 15852, and *A. flavus* AUMC 15820, were applied in the evaluation of the antifungal and antibacterial traits of the myco-synthesized AgNPs.

### 2.2 Isolation of endophytic fungal strain

Healthy and mature leaves of dill were used to recover the endophytic fungus. A sodium hypochlorite solution (NaOCl) of 1.5% was used for surface sterilization of the dill leaves then distilled water was used to wash it twice. After that, small pieces of leaves were plated onto potato dextrose agar (PDA)/28 ± 2°C for 7 days. The emerging colony was purified and morpho-genetically identified (Ghasemi-Sardareh and Mohammadi, 2020).

#### 2.2.1 Morphological identification of the isolated fungal endophyte

The fungal strain was grown on Czapek’s agar (CZA) medium for 7 days. Then the fungal endophyte was identified based on preliminary morphological and cultural features and deposited at Assiut University Mycological Centre (AUMC), Egypt.

#### 2.2.2 Molecular identification of the isolated fungal endophyte

The Patho-gene-spin DNA/RNA extraction kit purchased from Intron Biotechnology Company; Korea was used for DNA extraction. Fungal DNA was then undergone PCR and sequencing and analyzed using NCBI website (White et al., 1990). Finally, the phylogenetic analysis was generated using MegAlign (DNA Star) software (version 5.05).

### 2.3 Biosynthesis of AgNPs

**Figure 1:**
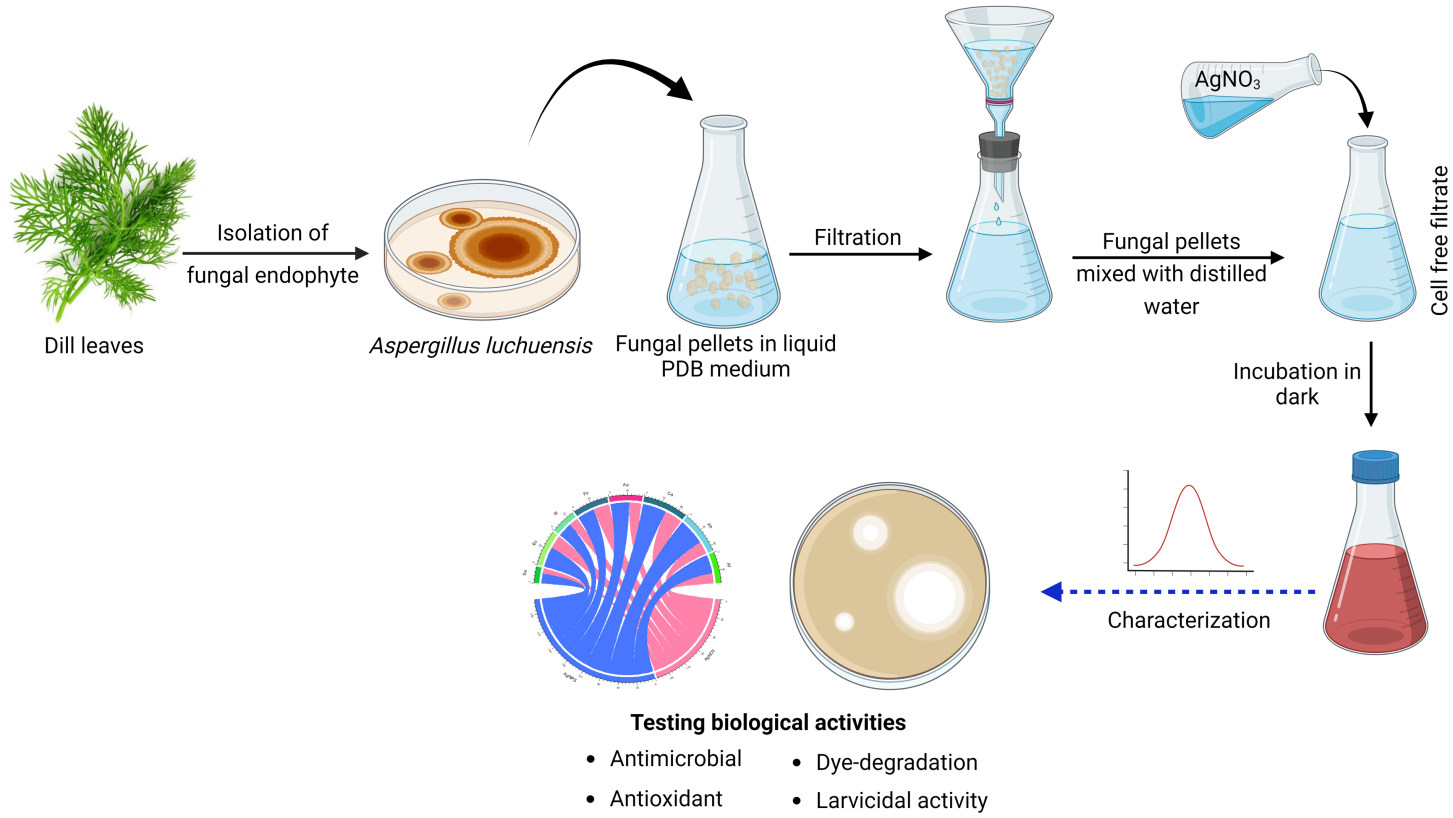
Graphical representation of workflow chart for mycosynthesis of silver nanoparticles and their applications.

AgNPs were produced based on the technique described by Gudikandula et al. (2017). *A. luchuensis* was grown on potato dextrose broth medium, followed by fungal pellets separation by filtration and aseptically washed with sterile distilled water (D.W). Twenty grams of pellets were homogenized in D.W. and incubated for 3 days at 25°C in under shaking. After that, using a Whatman filter, a CFF was harvested and used for nanoparticle synthesis. Both CFF and AgNO_3_ (1 mM) were equally combined (1:1 ratio) within the shaking incubator at 200 rpm, 25°C. The reddish-brown color formation confirmed that AgNPs had been synthesized (Eid et al., 2020). The control (aqueous solution of AgNO_3_ and cell-free filtrate without AgNO_3_) was performed under the same circumstances as the experiment (Figure 1).

### 2.4 Characterization of myco-synthesized AgNPs

The reduction of AgNO_3_ by metabolites of *A. luchuensis* was indicated by its color change. By detecting surface plasmon resonance characterized for AgNP at wavelengths from 300 to 800 nm, this absorbance was determined by a DS5 Dual Beam UV–Vis Spectrophotometer (DS5, Edinburgh, UK). Transmission electron microscopy (TEM; JEOL-JEM-1400, Tokyo, Japan) was employed to determine the particle and morphology of the synthesized AgNPs (Fouda et al., 2022a). Reduction and stabilization of AgNPs by different functional groups in the fungal filtrate were assessed by Fourier transform infrared spectroscopy (FTIR; Thermo Nicolet iS10 FTIR) at a scanning range of 400–4,000 cm^−1^. Furthermore, the purity of the nanoparticles was confirmed through energy-dispersive X-ray analysis (EDX) (JEOL, JSM-5500LV, and Tokyo, Japan). The charge on the AgNPs’ surface was determined by the Malvern Zetasizer apparatus (NanoZS, Malvern, United Kingdom).

### 2.5 Antimicrobial activity of AgNPs

Agar well diffusion assay was applied to determine the antimicrobial potential of AgNPs. Antibacterial activity was tested versus MDR Gram-positive bacteria *S. aureus* and Gram-negative bacteria *E. coli* and *S. typhi*, while antifungal activity was assessed using various pathogenic fungi, including *F. oxysporum*, *A. alternata*, *C. albucans*, *A. barasiliensis*, and *A. flavus*. Bacterial strains were grown in nutrient broth at 37°C for 24 h and adjusted to 0.5 as per McFarland standards. A 100 μL of bacterial suspension was spread on a nutrient agar plate. Three wells having a diameter of 7 mm were punched on the nutrient agar plate using a cork borer, and the synthesized AgNPs (5 mg/mL), AgNO_3_, and fungal filtrate were inoculated in each well. All the plates were incubated at 37°C for 24 h, and the antibacterial activity was evaluated by measuring the diameter of the inhibition zone. Similarly, the antifungal activity of AgNPs was performed by cultivating plates containing potato dextrose agar medium with the fungal strains; three wells with a diameter of 7 mm were made and inoculated with AgNPs, AgNO_3_, and the fungal filtrate (negative control). The plates were incubated at 28°C for 72 h, and the antifungal activity was evaluated by measuring the diameter of the inhibition zone (mm). All procedures were performed as previously described by Pawar and Patil (2020).

### 2.6 Assessment of antioxidant properties of AgNPs

Free radical scavenging activity of myco-synthesized AgNPs was determined by DPPH (2,2-diphenyl-1-picrylhydrazyl) assay as described by Salem et al. (2022) with minor modifications. In brief, a 0.1 mM solution of DPPH in ethanol was prepared. One mL of this solution was added to 3 mL of AgNPs at different concentrations (1,000, 500, 250, 125, 62.5, 31.25, 15.62, 7.81, and 3.9 μg/mL). The mixture was shaken vigorously and allowed to stand at room temperature for 30 min. Then, absorbance was measured at 517 nm by using a spectrophotometer (UV–VIS Milton Roy). The reference standard compound being used as ascorbic acid, and the experiment was done in triplicate DPPH scavenging effect (%) is calculated according to the following equation:


$$\begin{array}{l} \mathrm{DPPH scavenging effect}\;\left(\%\right)\\ \;=\frac{\mathrm{Absorbance}\;\mathrm{of}\;\mathrm{control}-\mathrm{Absorbance}\;\mathrm{of}\;\mathrm{sample}}{\mathrm{Absorbance}\;\mathrm{of}\;\mathrm{control}}\times 100 \end{array}$$

### 2.7 Assessment of photocatalytic activity of AgNPs

The catalytic potency of the AgNPs was estimated by the decomposition of safranin dye under light irradiation. Fifty mg of AgNPs (catalyst) was added to 100 mL of safranin dye (30 mg/L) followed by stirring (Hassanien et al., 2019). AgNPs’ photocatalytic efficacy was assessed according to Kalaimurugan et al. (2020) protocol. Based on the formula below, the photocatalytic dye degradation efficiency is calculated as follows:


$$D\;\left(\%\right)=\;\frac{A-B}{A}\;\times 100$$

where *A* reflects the solution’s beginning concentration and *B* final concentration of the safranin solution.

### 2.8 Mosquito larvicidal assay

#### 2.8.1 *Culex pipiens* colony

Mosquito (*Cx. Pipiens*) larvae were reared in enamel plates filled with de-chlorinated water and fed on fish food (Tetramin^®^). The colony was maintained at 27 ± 2°C, 70 ± 10% RH, and a 12:12 h (L/D) photoperiod. Adult mosquitoes fed on an 8–10% sucrose solution. Under the same laboratory conditions, larvae and adult females were continuously available for the experiments (M. B. M, 2013).

#### 2.8.2 Larvicidal activity

The larvicidal activity of *A. luchuensis* cell filtrate, spores suspension, and biosynthesized AgNPs was tested against 3^rd^ instar larvae of *Cx. pipiens* (World Health Organization, 2005). Different concentrations of each treatment in deionized water with 20 larvae of the 3^rd^ instar larvae. Bioassays were done at six concentrations (0.3, 0.6, 1.2, 1.5, 1.8, and 2.1 ppm). Control treatments were carried out with dechlorinated water only. The experiments were repeated five times. All mortalities were recorded at 24, 48, and 72 h post-treatment (PT).

#### 2.8.3 Scanning electron microscopy (SEM)

To estimate the impact of myco-synthesized AgNPs on the *Cx. pipiens* larvae. Treated and control samples were maintained in 3% glutaraldehyde, then washed and fixed in osmium tetroxide for 2 h, afterwards, the samples were dehydrated in serious ascending of ethanol. The fixed samples were then dried, coated with gold, and examined with SEM (JEOl, JCM 7000 LV).

### 2.9 Data analysis

Using the PASW Statistics 2009 (SPSS version 22) software, data were analyzed using one-way ANOVA followed by Duncan’s post-hoc. The probit assay was also used to determine the lethal values. R Studio software (version 3.6.1)^1^ was used for data visualization.

## 3 Results

### 3.1 Fungal isolation and identification

**Figure 2:**
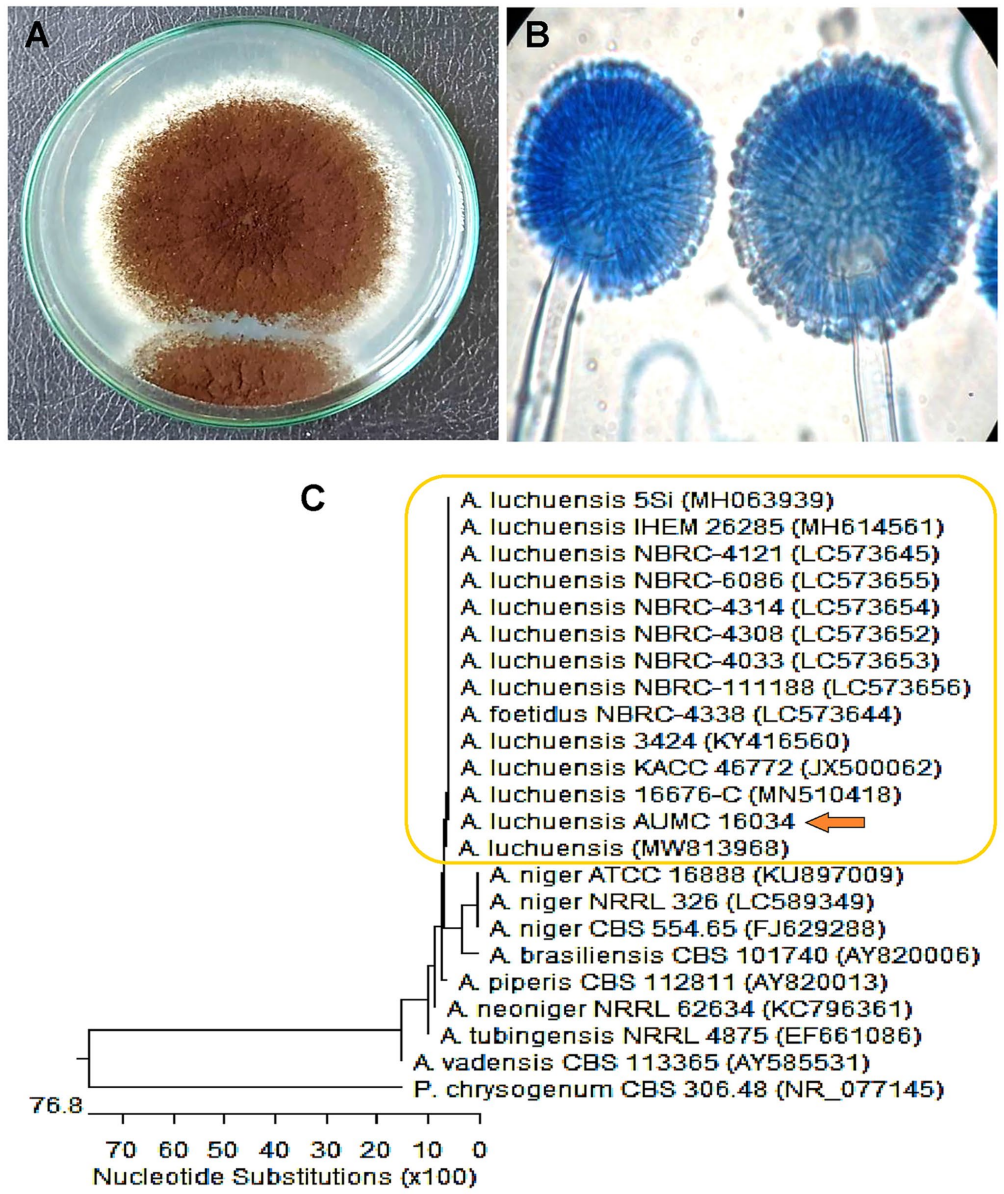
**(A)** Macro- and **(B)** micro-characteristics of *Aspergillus luchuensis* grown on Czapek’s-Dox agar at 30°C for 7 days. **(C)** Phylogenetic analysis of *A. luchuensis* and reference sequences conducted with MegAlign (DNA Star) software version 5.05 based on the neighbor-joining method.

The fungal strain was recovered from the leaf of Dill, and identification was performed by morphological characteristics and then confirmed by molecular identification. As a result of morphological identification, a fungal strain AUMC16034 had a brownish-black color with a growth rate of 5–7 cm in 7 days on Czapek’s agar (CZA) medium, as shown in Figure 2A. Furthermore, the conidial head is usually biseriate, the conidiophore is hyaline, thick-walled, and unbranched, and the conidia are globose, smooth, or slightly roughened (Figure 2B). The morphological identity was further confirmed based on sequencing data, which ascertained their taxonomic positions. The phylogenetic analysis shown in Figure 2C revealed that the fungal strain AUMC16034 exhibited similarity (99.82%) with *A. luchuensis*. Moreover, the sequence of *A. luchuensis* was submitted to Genbank under the accession number (PP315916).

### 3.2 Characterization of myco-synthesized AgNPs (UV–vis spectra, TEM and FTIR, EDX, and zeta potential analyses)

**Figure 3:**
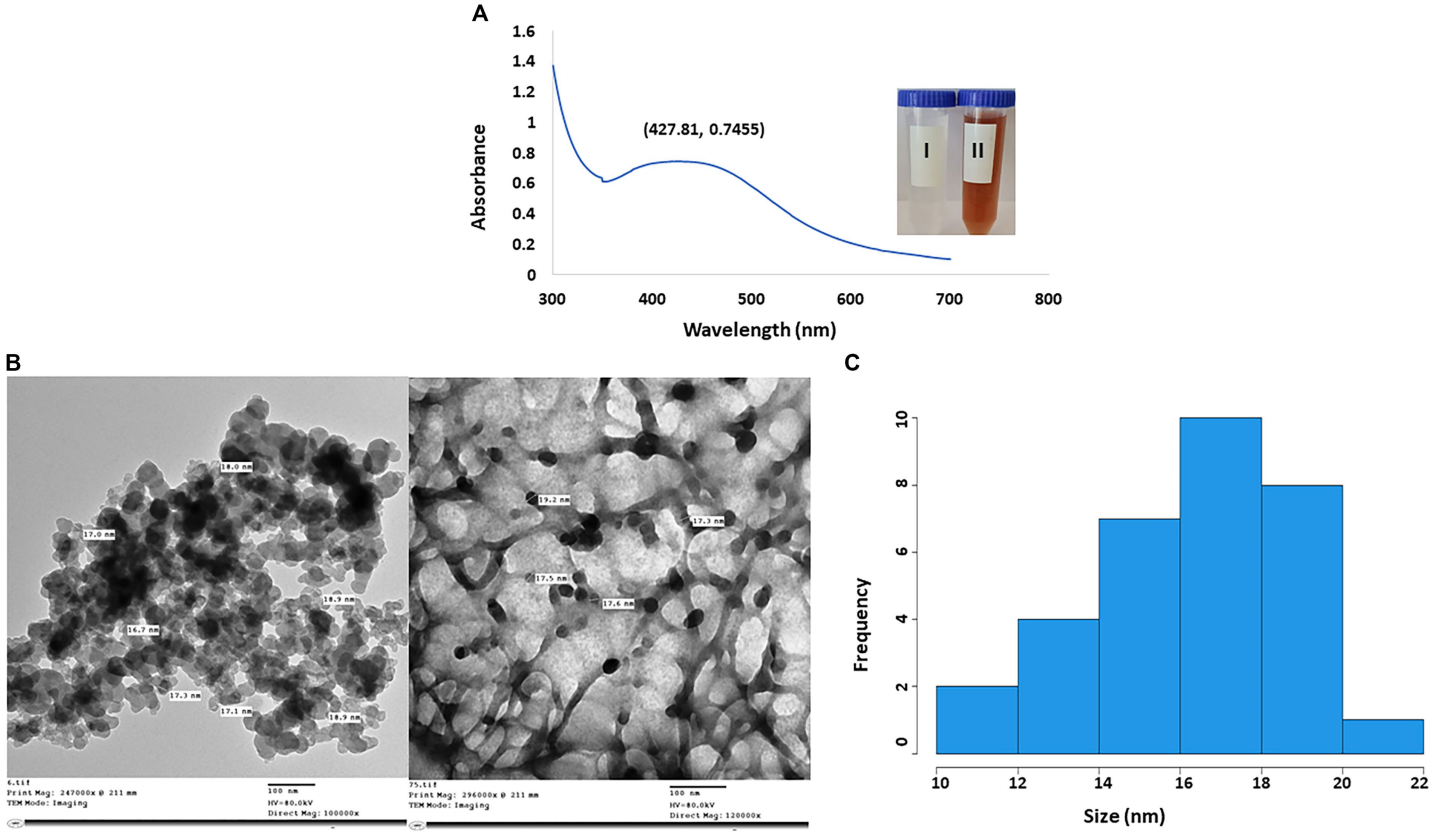
**(A)** UV-visible spectra of AgNPs synthesized by *Aspergillus luchuensis* (I) color of cell-free filtrate; (II) brown color due to AgNPs. **(B)** TEM image with a scale bar of 100 nm. **(C)** Histogram showing the size distribution based on the TEM image of myco-synthesized AgNPs from *A. luchuensis.*

The efficacy of fungal metabolites in the creation of AgNPs was assessed by the color transformation of the reaction mix to reddish brown. In Figure 3A, the highest peak of AgNPs was seen at 427.81 nm. Moreover, according to TEM micrographs, the CFF of *A. luchuensis* had the ability to fabricate AgNPs in a sphere shape without aggregation. The size of the myco-synthesized AgNPs was 16–18 nm (Figures 3B,C).

**Figure 4:**
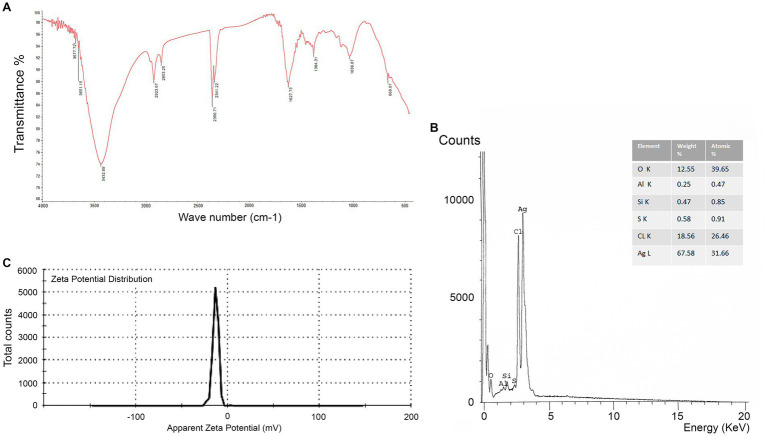
**(A)** FTIR spectrum showing associated functional groups in myco-synthesized AgNPs. **(B)** The EDX spectrum of AgNPs appears at the expected position of 3.0 keV. **(C)** Zeta potential of AgNPs synthesized by *Aspergillus luchuensis.*

By using FTIR, it was possible to examine the different functional groups found in the filtrate and their functions in the reduction, capping, and stabilization of AgNPs (Figure 4A). The peak formed at 3432.69 cm^−1^ could be attributed to the N-H and O-H overlapping stretching vibrations. The absorption bands observed at 2923.67 cm^−1^ and 2853.25 cm^−1^, respectively, can be ascribed to the O-H and C-H stretching vibrations of alcohol, carboxylic, and alkane groups. The peak creation at 2360.71 cm-1 verified the existence of S-H stretching. The presence of the peak at 2341.22 cm^−1^ is related to the O=C=O stretching of CO_2_ that is adsorbed onto the surface of proteins. The peak at 1627.73 cm^−1^ signified the C=O of polysaccharide moieties. The peak formed at 1384.41 cm^−1^ and 1023.13 cm^−1^ confirms the presence of S=O stretching. The peak at 668.97 cm^−1^ is C-Br stretching. Further, as illustrated in Figure 4B, the EDX spectrum exhibited a strong signal at 3 keV that was attributed to the SPR of Ag nanocrystals (67.58%) and weaker signals from Al (0.25%), Si (0.47%), S (0.58%), CL (18.65%), and O (12.55%) atoms. Next, the negative zeta potential (−13.2 mV) revealed the stability of AgNPs (Figure 4C).

### 3.3 Antimicrobial efficacy of AgNPs from *A. luchuensis*

**Figure 5:**
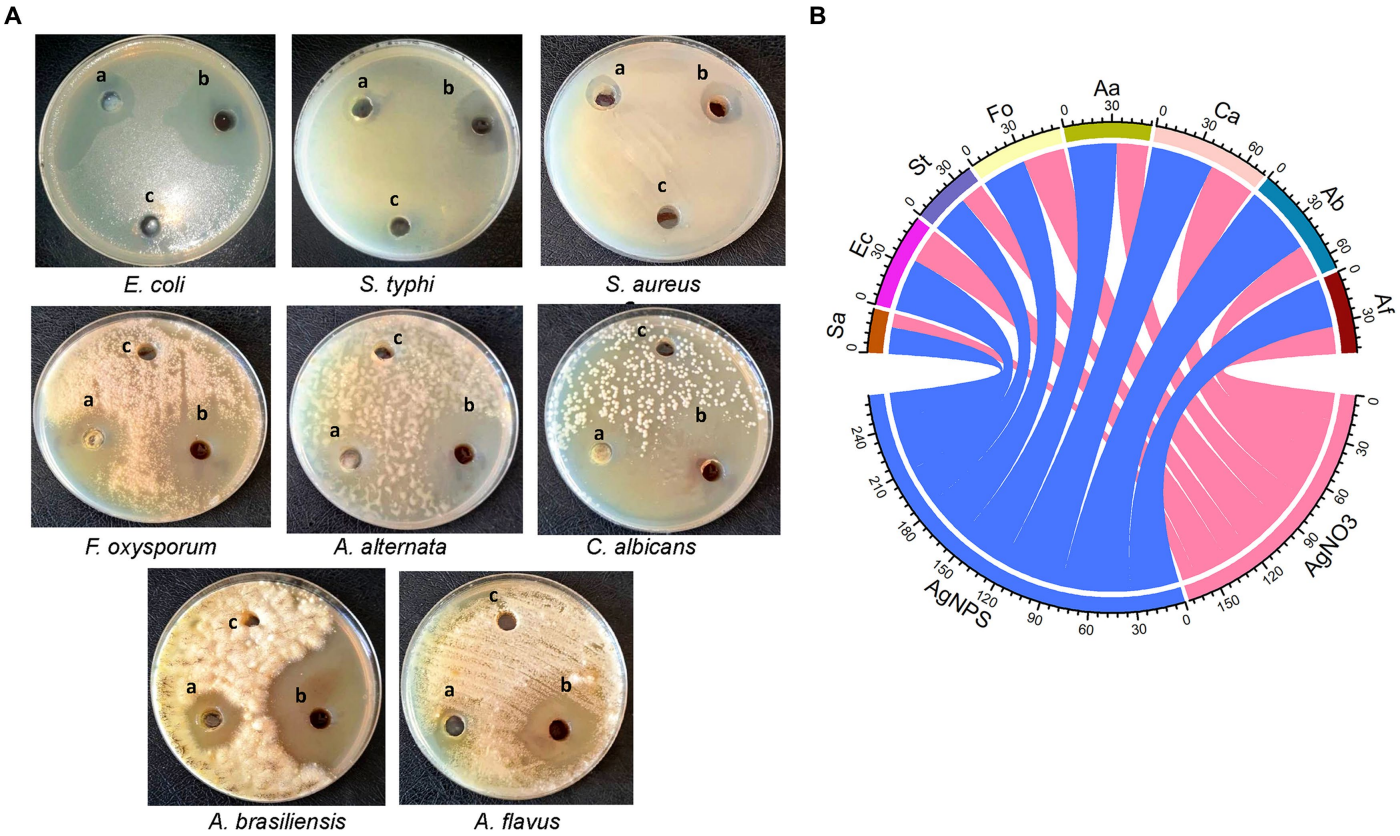
**(A)** Diameter inhibition zone (mm) of different pathogenic microorganisms treated with (A) AgNO_3_; (B) AgNPs; and (C) cell-free extract. **(B)** Chord diagram showing antimicrobial activity of AgNPs synthesized from *Aspergillus luchuensis* versus tested microorganisms. Sa, *S. aureus*; Ec, *E. coli*; St, *S. typhi*; Fo, *F. oxysporum*; Aa, *A. alternate*; Ca, *C. albicans*; Ab, *A. brasinsilles*; Af, *A. flavus.*

In the current work, the antimicrobial efficacy of myco-synthesized AgNPs was assessed versus a number of pathogenic microorganisms using the agar well diffusion technique (Figure 5A). Data revealed that AgNPs were effective versus all tested microorganisms as compared to cell-free filtrate and AgNO_3_ (Figure 5B). The highest growth inhibition (47 ± 0.17 mm) was recorded in *A. brasinsilles*, followed by *C. albicans* (42.1 ± 1.06 mm). Moreover, AgNPs had promising antibacterial efficacy versus all tested bacteria. Our data revealed that the maximum inhibitory activity was displayed in *E. coli* (35.3 ± 0.04 mm), followed by *S. typhi* (25.3 ± 0.17 mm). While the lowest growth inhibition (18 ± 0.05 mm) was exhibited against *S. aureus*.

### 3.4 Antioxidant activity of AgNPs fabricated by *A. luchuensis*

**Figure 6:**
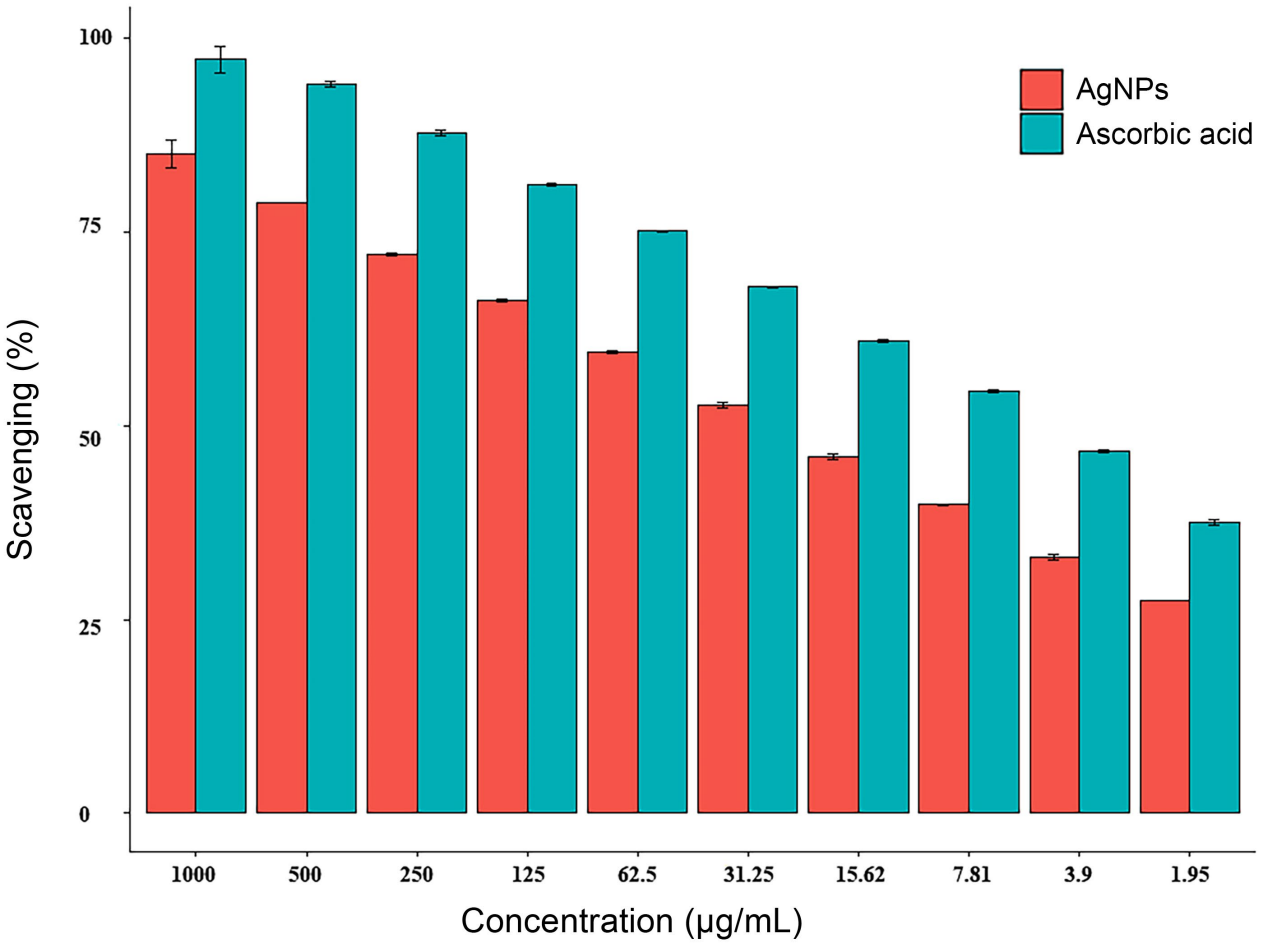
Antioxidant activity of AgNPs synthesized by *Aspergillus luchuensis.* Where, ascorbic acid served as a positive control. Data are expressed as means ± SD.

By the DPPH (2,2-diphenyl-1-picrylhydrazyl) assay, the radical scavenging activity of AgNPs was found to be dose-dependent, increased from 33.0 to 85.1% with increasing concentrations from 3.9 to 1,000 μg/mL as depicted in Figure 6. The IC_50_ of 23.11 g/mL and 5.43 g/mL for AgNPs and ascorbic acid (control) were observed, respectively.

### 3.5 Photocatalytic activity of myco-synthesized AgNPs

**Figure 7:**
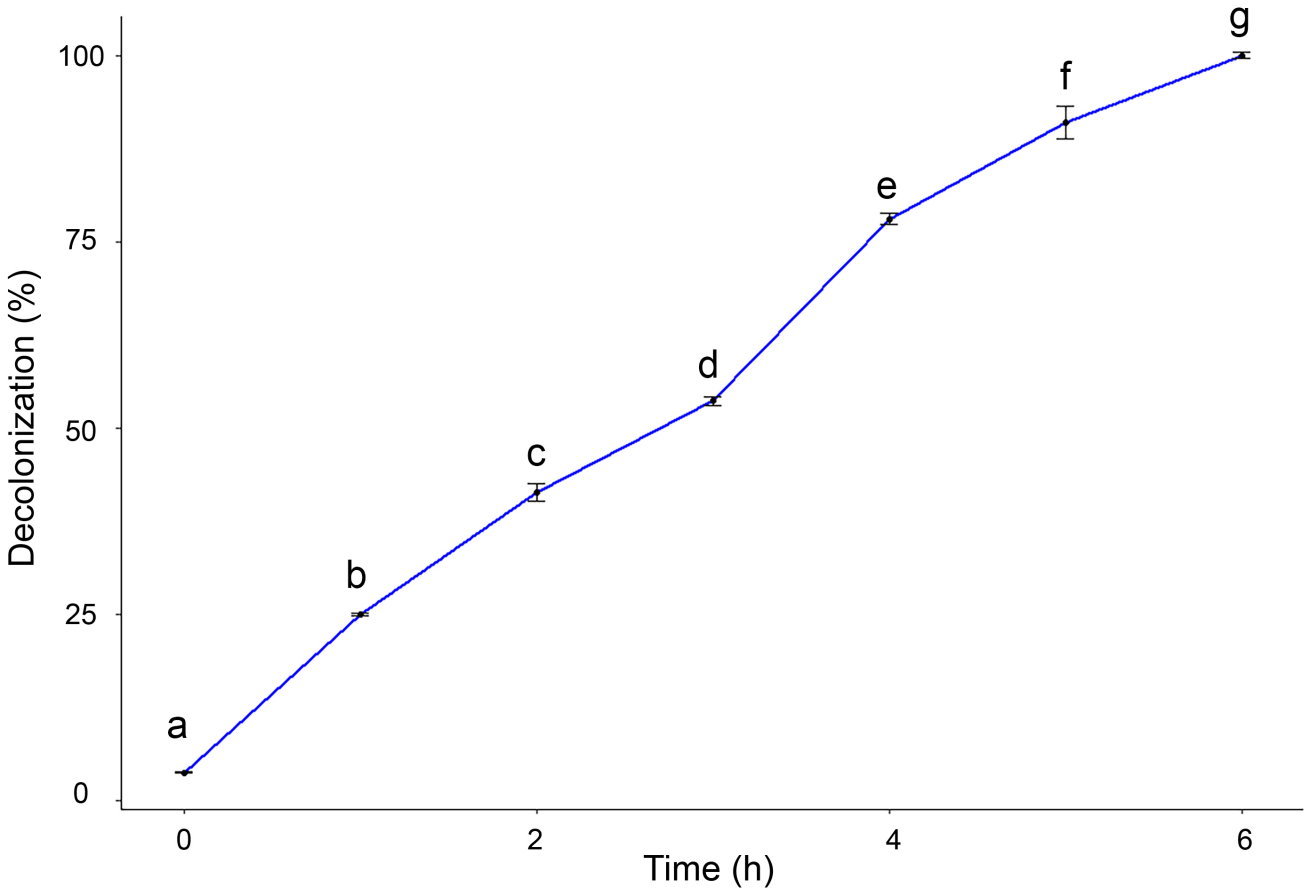
Degradation efficacy of safranin dye by AgNPs at different time under light irradiation condition. Data are expressed as means ± SD. Different small letters indicate significant difference at each time point (*p* < 0.01).

The catalytic efficacy of AgNPs was assessed by the degradation of safranin dye. The potential decolorization effects of AgNPs on safranin dye were examined at different contact times. Results clarified that by increasing exposure time, the degradation percentage of safranin dye increased. The maximum decolorization percentage (100%) was observed after 6 h under light irradiation, as shown in Figure 7.

### 3.6 Mosquito larvicidal activity

In the current work, the larvicidal potential of *A. luchuensis* cell filtrate, spores suspensions, and biosynthesized AgNPs were screened versus 3^rd^ instar larvae of *Cx. pipiens*. Data clarified that AgNPs fabricated by *A. luchuensis* had more toxic effects versus *Cx. pipiens* larvae than spore suspension and fungal cell filtrate. The MO % at 24 h PT of *Cx. pipiens* with log 2.1 ppm reached 98, 31 and 11% (Table 1) with LC_50_ = 1.55, 5.43, and 7.65 ppm for AgNPs, spore suspensions, and *A. luchuensis* cell filtrate, respectively (Table 2); whereas those of AgNPs were 100, 60, and 18 (MO %) with LC_50_ values = 0.89, 2.17, and 6.46 ppm after 42 h PT, respectively. Data presented in Tables 1, 2, cleared that the LC_50_ values of AgNPs (0.65 ppm) were more effective than those of *A. luchuensis* spore suspensions and CFF (1.10 ppm and 5.32 ppm), respectively, versus the 3^rd^ larval instar of *Cx. pipiens*, 72 h PT.

**Table 1:** Efficacy of *A. luchuensis* cell free filtrate, spore suspensions, and AgNPs on *Culex pipiens* larval mortality.

Treatment	Conc. (ppm)	Mortality (h)		
		24	48	72
Cell free filtrate	0.0	0 ± 0^eA^	0 ± 0^fA^	0 ± 0^gA^
	0.3	0 ± 0^eB^	0 ± 0^fB^	4 ± 1.00^fA^
	0.6	1 ± 1.00^deC^	3 ± 1.22^eB^	7 ± 1.22^eA^
	1.2	2 ± 1.22^dC^	7 ± 1.22^dB^	11 ± 1.00^dA^
	1.5	5 ± 1.58^cC^	9 ± 1.00^cB^	17 ± 1.22^cA^
	1.8	8 ± 2.00^bC^	13 ± 2.00^bB^	20 ± 2.24^bA^
	2.1	11 ± 2.45^aC^	18 ± 1.22^aB^	35 ± 2.74^aA^
Spore suspensions	0.0	0 ± 0^gA^	0 ± 0^gA^	0 ± 0^gA^
	0.3	4 ± 1.00^fC^	8 ± 1.00^fB^	11 ± 1.87^fA^
	0.6	9 ± 1.00^eC^	15 ± 1.58^eB^	25 ± 1.87^eA^
	1.2	12 ± 1.22^dC^	22 ± 1.22^dB^	36 ± 2.55^dA^
	1.5	18 ± 2.00^cC^	33 ± 3.74^cB^	60 ± 1.87^cA^
	1.8	26 ± 1.87^bC^	45 ± 2.92^bB^	75 ± 3.67^bA^
	2.1	31 ± 1.87^aC^	60 ± 2.74^aB^	90 ± 0.00^aA^
AgNPs	0.0	0 ± 0^gA^	0 ± 0^gA^	0 ± 0^gA^
	0.3	8 ± 1.22^fC^	15 ± 1.22^fB^	22 ± 4.36^fA^
	0.6	14 ± 1.87^eC^	30 ± 1.87^eB^	42 ± 2.55^eA^
	1.2	30 ± 1.87^dC^	48 ± 1.87^dB^	63 ± 2.55^dA^
	1.5	40 ± 2.92^cC^	65 ± 2.92^cB^	84 ± 2.92^cA^
	1.8	55 ± 4.58^bC^	85 ± 4.58^bB^	100 ± 0.00^bA^
	2.1	98 ± 4.64^aA^	100 ± 0.00^aA^	100 ± 0.00^aA^

Data are presented as mean ± SD. Means within the same column or the same row with the different letters are considered significant at *p* < 0.05.

**Table 2:** Lethal concentrations (ppm) of *A. luchuensis* cell free filtrate, spore suspensions, and AgNPs against *Culex pipiens*.

Treatment	Time (h)	LC_50_ (Low-Up)	LC_90_ (Low-Up)	Slope ± SE	Chi (Sig.)
Cell free filtrate	24	7.65 (4.15–66.28)	26.72 (9.38–125.41)	2.312 ± 0.690	1.031 (0.904)
	48	6.46 (4.04–20.50)	28.53 (11.52–281.32)	1.9872 ± 0.441	1.120 (0.891)
	72	5.32 (3.57–11.80)	35.19 (14.70–218.01)	1.564 ± 0.282	7.237 (0.123)
Spore suspensions	24	5.43 (3.59–12.48)	40.60 (16.14–280.68)	1.466 ± 0.267	3.283 (0.511)
	48	2.17 (1.76–4.93)	10.45 (10.20–115.54)	1.876 ± 0.232	12.138 (0.016)
	72	1.10 (0.68–1.64)	3.32 (3.36–10.84)	2.655 ± 0.223	26.665 (0.000)
AgNPs	24	1.55 (1.14–2.78)	5.20 (5.07–28.90)	2.438 ± 0.241	20.975 (0.000)
	48	0.89 (0.48–1.29)	2.57 (2.51–8.06)	2.782 ± 0.217	33.518 (0.000)
	72	0.65 (0.36–0.86)	1.70 (1.38–3.46)	3.046 ± 0.211	31.766 (0.000)

### 3.7 Scanning electron microscopy (SEM)

**Figure 8:**
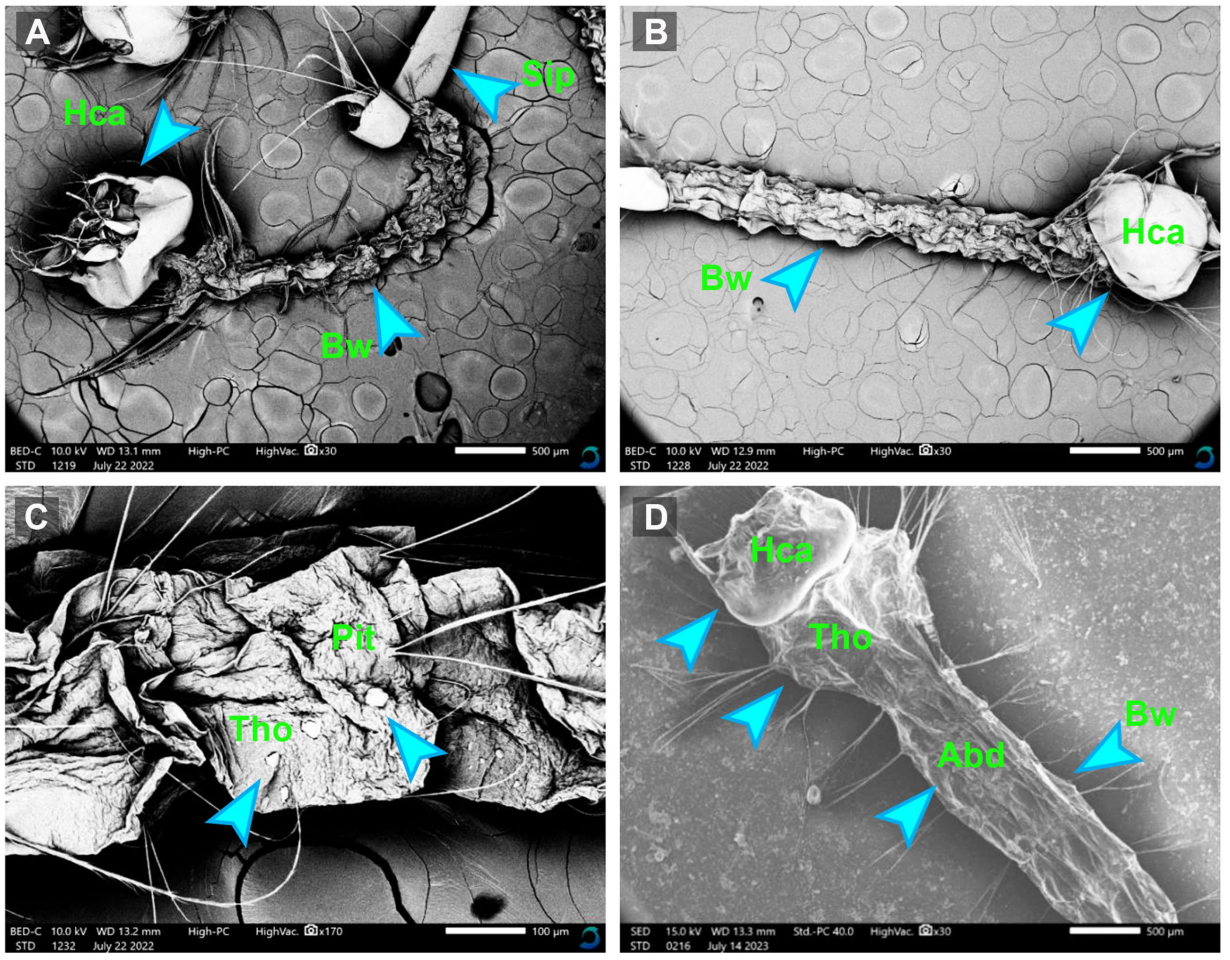
The SEM micrographs of treated *Culex pipiens* larvae **(A–C)** by myco-synthesized AgNPs for 48 h incubation period and untreated mosquito larvae **(D)**. Hca, head capsule; Tho, thorax area; Abd, abdominal segment; Bw, body wall; Sip, siphon.

The morphological alternations in the mosquito larvae treated and untreated with myco-synthesized AgNPs were assessed by the scanning electron microscope. After exposing mosquito larvae to myco-synthesized AgNPs, the larval body became completely weak compared to the control. Moreover, the results clarified that the larval tissues became damaged and suffered from major changes including tearing, the disappearance of the epithelial layer, and flattening of the tissue, as shown in Figure 8.

## 4 Discussion

Researchers are paying more attention to AgNPs because of their promising activities in numerous fields (Albulym et al., 2021). Consequently, attempts have increased to synthesize AgNPs using different methods. To overcome the limitations of physical and chemical and approaches, it is preferable to use plants, fungi, yeasts, bacteria, and actinomycetes for the green production of metallic NPs (Salem and Fouda, 2021). The myco-synthesized nanoparticles provide a number of benefits versus bacteria since most fungi are simple to handle, require few nutrients, have strong wall-binding capacities, and can take up metals intracellularly (Sanghi and Verma, 2009).

The production of AgNPs from fungal endophytes has a significant application in the biomedical and pharmaceutical fields. Endophytic fungi are biologically significant microorganisms because they can produce novel medicinal compounds, biocontrol agents, and other beneficial products, according to the study’s findings (Kathiresan et al., 2016). In this investigation, the endophytic fungal strain *A. luchuensis* recovered from the leaves of Dill was assessed for the inhibition of Ag^+^ ions extracellularly to form NPs. The synthesis of AgNPs with fungal filtrate was verified using the SPR; herein, the color transformed from colorless to reddish brown, indicating AgNPs had been synthesized. A color shift resulted from the excitement in the SPR of NPs (Salem et al., 2020). There was a maximum Plasmon absorption band indicating that the AgNPs are round. In this regard, Abdel-Hadi et al. (2014) mentioned that myco-synthesized AgNPs by *A. terreus* displayed a SPR band at 425 nm. Moreover, the absorption spectra of AgNP synthesized from the endophytic fungus *A. versicolor* showed a peak at 429 nm (Netala et al., 2016). In the same data frame, Osorio-Echavarría et al. (2021) recorded that the absorption spectrum of AgNPs was detected at 430 nm by using fungus *Anamorphous bjerkandera* sp. On the other hand, Devi and Joshi (2015) reported that biosynthesized AgNPs from the endophytic fungus *Cladosporium cladosporioides* exhibited an absorption spectrum of 440 nm. According to the previous reports, a successful synthesis of AgNPs is indicated by the noted SPR peak within the scope of 400–460 nm (Dong et al., 2017).

According to the current investigation, the metabolic products of *A. luchuensis* have the ability to reduce or “cap” AgNO_3_ and make spherical AgNPs that are well distributed. In addition, Elsayed et al. (2018) synthesized spherical AgNPs with sizes range of 3–20 nm by using *A. niger* NRC1731 biomass filtrate. Furthermore, the particle sizes of AgNPs synthesized from *A. fumigatus* range from 5 to 25 nm (Bhainsa and D'souza, 2006). It was reported that the white-rot fungus *Bjerkandera* sp. was able to generate spherical AgNPs with a size range of 10–30 nm (Osorio-Echavarría et al., 2021). In contrast, Li et al. (2011) was able to effectively generate well-dispersed, spherical AgNPs with a mean size of 4.3 nm by using the metabolites of *A. terreus*.

Furthermore, the FTIR results assured the existence of bioactive compounds, including amino acids, carboxylates, alkenes, and carbohydrates, which have previously been suggested as potential reducing agents for the generation of metal and metal oxide NPs (Fouda et al., 2022b). According to Vanaja et al. (2013), the amine and carboxylic groups are functional ingredients implicated in the reduction of Ag^+^ ions. The FTIR absorption peak appears to be related to the reduction of Ag^+^ ions, indicating the chemical purity of AgNPs.

The charge that is found on the nanoparticles’ surface determines the electrostatic repulsive force between them. In the current work, the obtained data from the zeta potential assay indicated that myco-synthesized AgNPs have a negative charge. This agrees with the data obtained from Balakumaran et al. (2016), who found the −ve zeta potential of myco-synthesized AgNPs by *A. terreus*. This −ve value might be due to the existence of the reducing contents in the fungal cell filtrate, which exhibits electrostatic forces in green-synthesized NPs (Nasrollahzadeh and Sajadi, 2016).

It has been reported that secondary metabolites are necessary for reducing AgNO_3_ to AgNPs and are vital for optimizing the morphology and stability of the AgNPs. Furthermore, the presence of metallic Ag was emphasized by EDX; thereby, the AgNPs biosynthesis was successfully liberated. EDX spectrum shows other peaks which indicates that biomolecules were bound to the surface of AgNPs during the process (Ravichandran et al., 2019). It was emphasized that metallic Ag nanocrystals typically appear at 3.0 keV in EDAX analysis because of their SPR (Devi and Joshi, 2015). In keeping with the consequences, Othman et al. (2019) mentioned that AgNPs synthesized from *A. fumigatus* displayed strong signal energy peaks similar to ours.

Antioxidants are effective in preventing free radicals formation, which causes several disorders (Dharmaraja, 2017). Antioxidants have been considered therapeutic agents due to their anticancer, antibacterial, anti-inflammatory, anti-mutagenic, and anti-atherosclerotic traits. The DPPH assay indicated that AgNPs had strong antioxidant properties, and their ability to scavenge radicals increased as concentration increased. Our findings validate previous reports regarding the antioxidant characteristics of Ag-NPs (Hulikere and Joshi, 2019).

Nearly most of pathogenic microorganisms have grown resistant to every kind of antibiotic that is currently used (Teixeira et al., 2018). Previously, the antimicrobial activity of the biosynthesized AgNPs was formerly recorded (Elbahnasawy et al., 2021; Sudarsan et al., 2021). In the present work, the inhibitory effect of myco-synthesized AgNPs was tested against a variety of pathogenic isolates using the agar well diffusion assay. Results indicated that AgNPs fabricated by *A. luchuensis* had promising antimicrobial potential versus pathogenic bacterial and fungal strains. AgNPs are typically observed to be more efficient against Gram-negative bacteria as opposed to Gram-positive bacteria that could be due to differences in the composition of the cell wall. Gram-negative bacteria have a thin layer of peptidoglycan that allows the Ag^+^ ions to enter the cell and a lipopolysaccharide layer with a negative charge that attracts Ag^+^ ions, causing enhanced absorption and breaking down the cell wall (Vanaja et al., 2013).

Our findings are in coincidence with the data obtained by Netala et al. (2016), who reported that AgNPs synthesized from *Cladosporium* displayed strong inhibition to Gram-negative compared to Gram-positive bacteria. Additionally, Shahzad et al. (2019) found that synthesized AgNPs displayed antibacterial potential versus multidrug-resistant bacterial strains, notably *K. pneumoniae* BTCB04, *Acinetobacter* BTCB05, *P. aeruginosa* BTCB01, and *E. coli* BTCB03, while a maximum 7-fold increase was observed with *Acinetobacter* BTCB05. Mahitha et al. (2011) demonstrated the antibacterial efficacy of AgNPs against Gram +ve (*B. subtilis*, *S. aureus*) and Gram −ve (*E. coli*, *K. pneumoniae*) bacteria. Huang et al. (2007) documented that AgNPs ranging in size from 10 to 25 nm are potent against pathogenic microbes. Shrivastava et al. (2007) performed research on the antimicrobial potential of AgNPs. It was reported that AgNPs from *Alternaria* sp. are effective against MDR strains (Baker et al., 2021).

Moreover, our data showed that the myco-synthesized AgNPs displayed antifungal potential, as estimated by the agar-well diffusion assay, versus *A. brasinsilles*, *C. albicans*, *A. alternata*, *A. flavus*, and *F. oxysprum*, respectively. Our data are in parallel with the those explained by Wang et al. (2021), who tested the antifungal efficacy of myco-synthesized AgNPs from *A. sydowii* versus many clinical pathogenic fungi, including *C. albicans*, *C. glabrata*, *C. parapsilosis*, *C. tropicalis, F. solani*, *F. moniliforme*, *F. oxysporum*, *A. favus*, *A. fumigatus*, *A. terreus*, *Sporothrix schenckii*, and *C. neoformans*. The biosynthesized AgNPs, from *A. fumigatus* DSM819 CFF, displayed antimicrobial potential toward the pathogenic microorganisms. In other studies by Baker et al. (2021) they evaluated the antifungal activity of AgNPs from *Alternaria* sp. versus fungal strains, including *A. niger, A. flavus*, *F. oxysporum,* and *T. viridens*.

**Figure 9:**
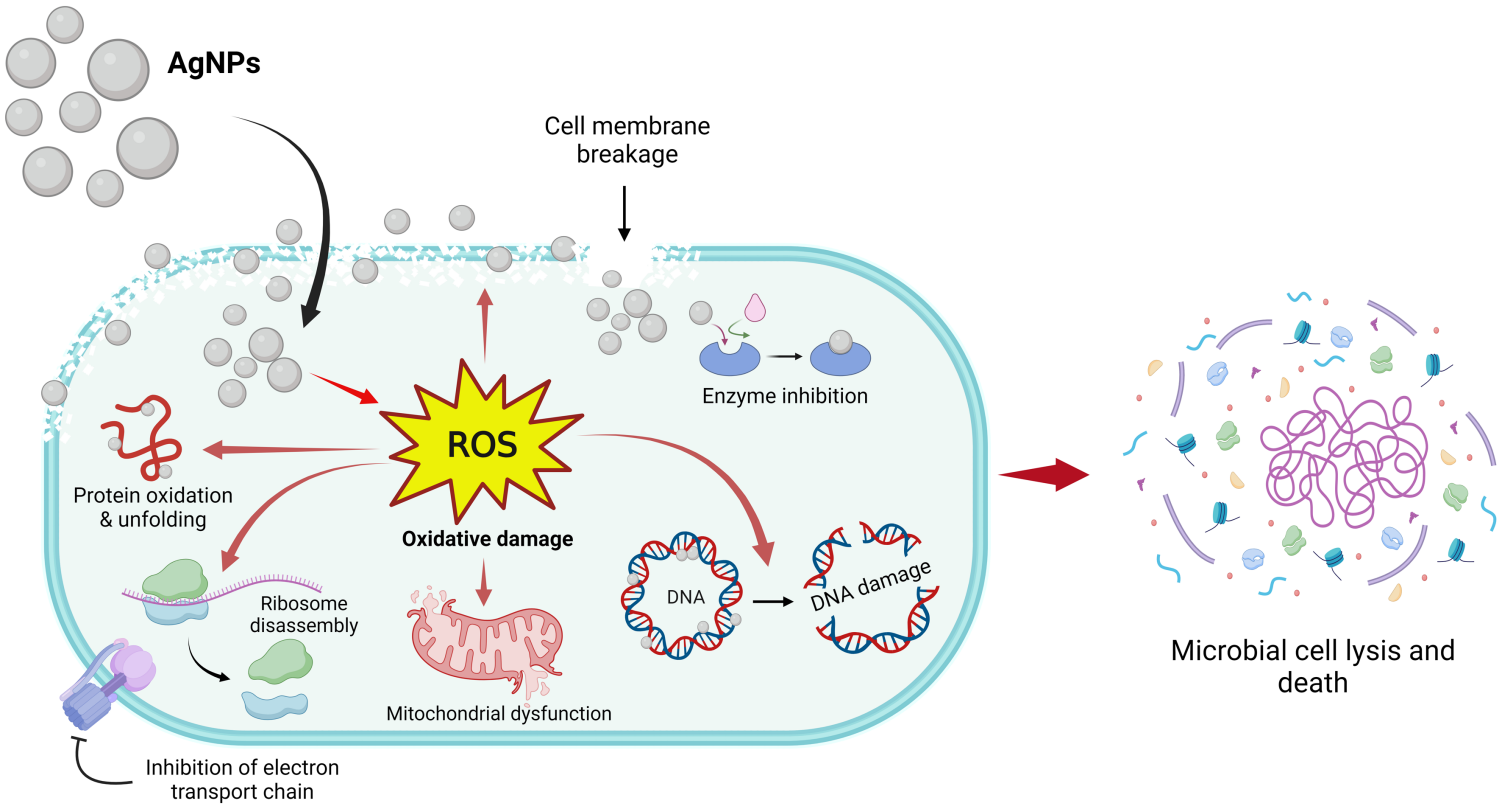
Antimicrobial mechanisms of AgNPs fabricated by *Aspergillus luchuensis*.

Based on the research findings, the suggested antimicrobial traits of AgNPs are depicted in Figure 9. AgNPs release Ag^+^ ions that gather on the microbial cell wall and plasma membrane and then in the cytoplasm involved in the formation of ROS inside the cell. ROS are the main agent for antimicrobial potential and involve inhibition of DNA synthesis, damage in the cell membrane, and constituent leakage, suppression of the production of proteins, mitochondrial dysfunction, and inhibition of the electron transport chain followed by cell lysis (Jain et al., 2021).

**Figure 10:**
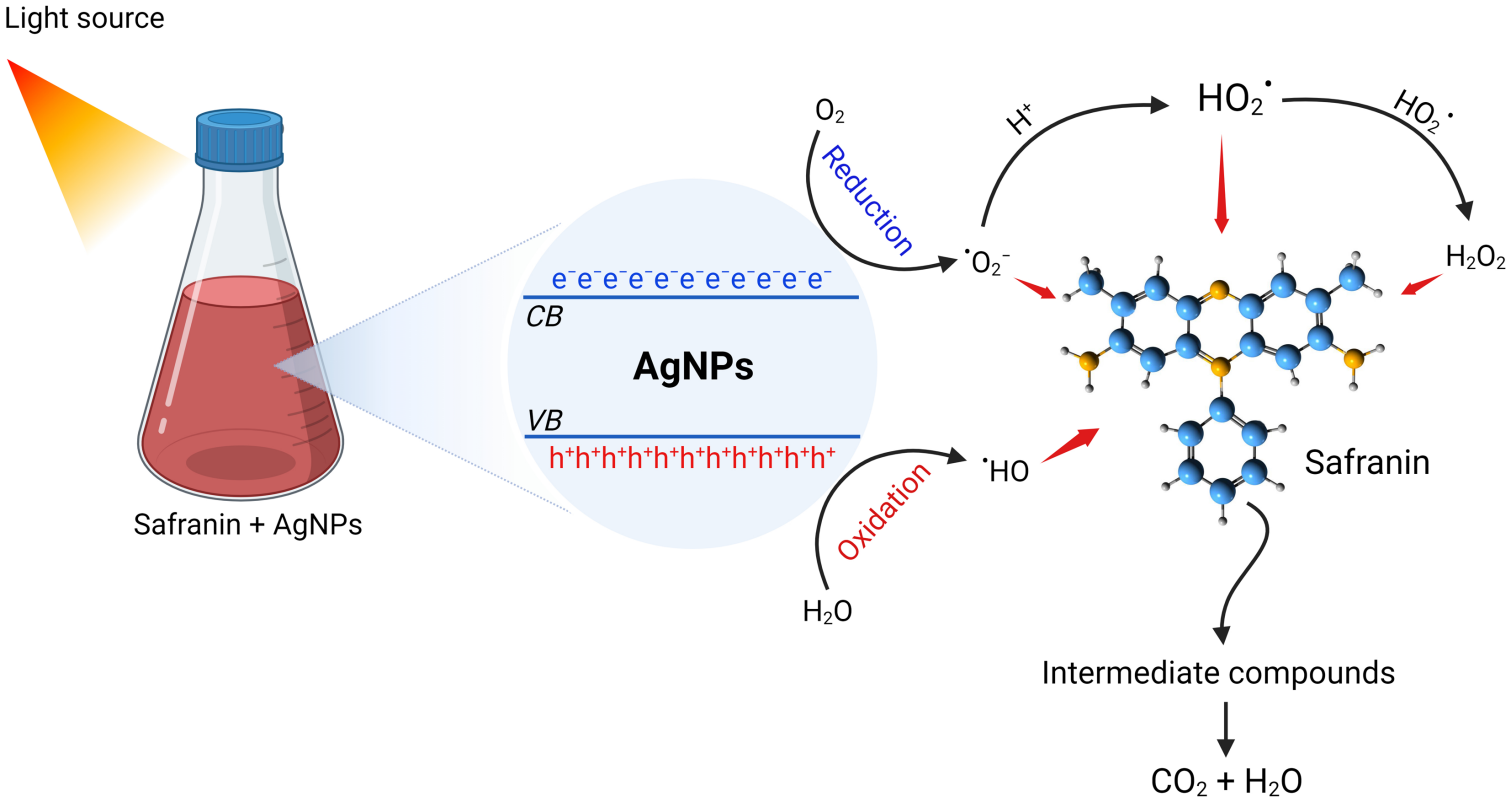
Photocatalytic degradation mechanism of safranin dye by AgNPs synthesized from *Aspergillus luchuensis.*

Bhakya et al. (2015) stated that nanocatalysts’ surface area is the key component that determines how well they work as catalysts, since the reactions happen on the surface. Smaller sizes and a high surface area are characteristics of nanocatalysts. As a result, reducing the size of the NPs will enhance catalytic (Saied et al., 2021). The catalytic property of AgNPs is indicated by the degradation of safranin dye. Under light irradiation, the catalytic effectiveness was attained at various contact times. It was found that the decolorization percentage enhanced along with the time increase. Our findings are in agreement with (Fouda et al., 2022b), who showed that AgNPs from *A. flavus* exhibited high photocatalytic efficacy in degrading MB dye. Furthermore, AgNPs synthesized using *Saussurea costus* extract effectively degraded the safranin dye (Abd El-Aziz et al., 2021). After exposure to light, electrons are triggered from the valence band (VB) to the conducting band (CB), forming electron–hole pairs [Ag (e^−^_CB_ and h^+^_VB_)]. The h^+^_VB_ reacts with H_2_O, creating hydroxyl radicals (^**•**^OH) and H^+^, while the e^−^_CB_ reduces O_2_, producing ^**•**^O_2_^−^ (superoxide radicals) and ^**•**^OOH (hydrogen peroxide radicals). Eventually, the active radical species (^**•**^OH, ^**•**^O_2_^−^, and ^•^OOH) reacted with safranin, contributing to increased dye degradation. Fouda et al. (2022b) claimed that the photocatalytic efficacy of NPs may be due to photogenerated holes (that undergo oxidation conditions to form hydroxyl radicals) and electrons that react with molecular oxygen to form oxygen anion radicals, resulting in a complete destruction of dyes to CO_2_, H_2_O, and small ions. The present work emphasized the potent antimicrobial and catalytic activity of myco-synthesized AgNPs. Consequently, the advantage of this phenomena is the ability to obtain wastewater that is devoid of microbes and dyes, allowing for safe reuse or release into the ecosystem (see Figure 10).

In the present investigation, the larvicidal potential of *A. luchuensis* cell-free filtrate, spores suspensions, and biosynthesized AgNPs were tested versus 3^rd^ instar larvae of *Cx. pipiens*. They showed low to strong insecticidal effects versus *Cx. pipiens*, after various intervals of exposure. Data revealed that biosynthesized AgNPs were the most efficient against *Cx. pipiens* larvae than fungal spore’s suspension and fungal cell-free filtrate. Microorganisms, like fungi, bacteria, and protozoa, are included in the biological systems used against mosquitoes and other medical pests (Hegazy et al., 2022; Katak et al., 2023). The present results indicate a positive relationship between mortality rate, biosynthesized AgNPs, and dose level, as stated by Sowndarya et al. (2017). In a similar work, the effectiveness of AgNPs synthesized from *F. oxysporum* against *Aedes* mosquito larvae was shown. The authors showed that *F. oxysporum* NPs had promising antibiological activity by killing *Aedes* larvae (Sumera et al., 2021). NPs’ larvicidal potential could be explained by the denaturation of sulfur-containing proteins and phosphorus-containing compounds, which results in the denaturation of organelles and enzymes. Consequently, cellular membrane permeability is reduced and ATP synthesis is reduced, leading to the death of cells and their lack of function (Shankar et al., 2004).

Only 5% of pesticides used worldwide are biopesticides, notwithstanding their effectiveness as insecticides (Kumar et al., 2021; Rakshit et al., 2021). But biopesticides are growing quickly and are expected to overtake chemical pesticides soon, with an average annual growth rate of 9–20% (Lahlali et al., 2022). This is because biopesticides have unique qualities that make them useful, such as not being harmful to the environment. Fungi have already been used in conjunction with other approaches to suppress vector mosquitoes (Cafarchia et al., 2022). In a recent study, antiparasitic activity was found in 152 of the tested fungi, which is 17.7%. *Aspergillus*, *Penicillium*, *Fusarium*, *Neocosmopora,* and *Thricoderma* had the strongest effects (Toghueo et al., 2019). Vivekanandhan et al. (2023) have documented that *A. niger* crude and purified extracellular extracts have larvicidal efficacy toward *Ae. Aegypti, An. stephensi,* and *Cx. quinquefasciatus*.

In addition, it becomes clear to us the power of AgNPs and their ability to improve the effectiveness of the biological component in killing mosquito larvae (Thelma and Balasubramanian, 2021; Shukla et al., 2022). Aside from their environmental friendliness, cost-effectiveness, and target-specific sensitivity, green synthetic nanoparticles have recently been at the forefront of efforts to develop a safe insecticide.

## 5 Conclusion

In the present work, AgNPs were synthesized in an eco-friendly manner by employing a biomass filtrate containing metabolites of endophytic fungus *A. luchuensis*. The synthesized AgNPs were characterized by UV–Vis spectrometer, FT-IR, TEM, EDX, and zeta potential. Furthermore, in the biological investigation, the biosynthesized AgNPs exhibited effective antibacterial activity against the studied multidrug-resistant bacteria as well as antifungal efficacy versus the pathogenic fungi. Also, it showed a strong antioxidant potential and their ability to scavenge radicals increased from 33.0 to 85.1% with increments in their concentration from 3.9 to 1,000 μg/mL. Additionally, myco-synthesized AgNPs displayed strong photocatalytic activity in the degradation of safranin dye. Surprisingly the biosynthesized AgNPs exhibited promising anti-insect properties against 3rd larval instar of *Cx. pipiens*. It is possible to deduce from the collected data the prospective applications of AgNPs synthesized using a green approach across multiple fields.

## Data availability statement

The data presented in the study are deposited in GenBank under the accession number (PP315916).

## Ethics statement

The insect study was approved by Ethics Committee of Faculty of Science, Benha University (Code: BUFS-REC-2024-105 Bot). The study was conducted in accordance with the local legislation and institutional requirements.

## Author contributions

RA: Conceptualization, Investigation, Methodology, Writing – original draft, Writing – review & editing. AE: Conceptualization, Investigation, Methodology, Writing – original draft, Writing – review & editing. ET: Conceptualization, Investigation, Methodology, Writing – original draft, Writing – review & editing. MB: Conceptualization, Investigation, Methodology, Writing – original draft, Writing – review & editing. HN: Data curation, Formal analysis, Software, Validation, Visualization, Writing – original draft, Writing – review & editing. AA: Conceptualization, Formal analysis, Investigation, Methodology, Software, Validation, Visualization, Writing – original draft, Writing – review & editing. ME-N: Conceptualization, Investigation, Methodology, Writing – original draft, Writing – review & editing. KA: Data curation, Formal analysis, Software, Validation, Visualization, Writing – original draft, Writing – review & editing. OM: Data curation, Formal analysis, Software, Validation, Visualization, Writing – original draft, Writing – review & editing. B-DI: Data curation, Formal analysis, Software, Validation, Visualization, Writing – original draft, Writing – review & editing. AK: Data curation, Formal analysis, Software, Validation, Visualization, Writing – review & editing. RA-S: Data curation, Formal analysis, Software, Validation, Visualization, Writing – review & editing. AS: Conceptualization, Investigation, Methodology, Writing – original draft, Writing – review & editing.

## Footnotes

^1^ https://www.R-project.org
